# Emotional Actions Are Coded via Two Mechanisms: With and without Identity Representation

**DOI:** 10.3389/fpsyg.2016.00693

**Published:** 2016-05-11

**Authors:** Joanna Wincenciak, Jennie Ingham, Tjeerd Jellema, Nick E. Barraclough

**Affiliations:** ^1^Department of Psychology, University of HullHull, UK; ^2^Department of Psychology, University of YorkYork, UK

**Keywords:** action perception, emotion, adaptation, social perception, social cognition

## Abstract

Accurate perception of an individual's identity and emotion derived from their actions and behavior is essential for successful social functioning. Here we determined the role of identity in the representation of emotional whole-body actions using visual adaptation paradigms. Participants adapted to actors performing different whole-body actions in a happy and sad fashion. Following adaptation subsequent neutral actions appeared to convey the opposite emotion. We demonstrate two different emotional action aftereffects showing distinctive adaptation characteristics. For one short-lived aftereffect, adaptation to the emotion expressed by an individual resulted in biases in the perception of the expression of emotion by other individuals, indicating an identity-independent representation of emotional actions. A second, longer lasting, aftereffect was observed where adaptation to the emotion expressed by an individual resulted in longer-term biases in the perception of the expressions of emotion only by the same individual; this indicated an additional identity-dependent representation of emotional actions. Together, the presence of these two aftereffects indicates the existence of two mechanisms for coding emotional actions, only one of which takes into account the actor's identity. The results that we observe might parallel processing of emotion from face and voice.

## Introduction

Information about the identity and emotional state of other individuals can be derived from a variety of social cues, including the face, the voice, the body, and whole body actions. The accurate perception of these cues underlies our ability to function successfully in a complex social environment. To date, models have been developed to explain how identity and emotion are derived from faces (Bruce and Young, 1986; Haxby et al., 2000) and voices (Belin et al., 2004; Campanella and Belin, 2007). However, our understanding of how we process identity and emotion derived from bodily actions is limited, despite the importance of action information in social cognition (de Gelder, 2006; de Gelder et al., 2010; Jellema and Perrett, 2012).

Early theories of face perception have argued that recognition of emotional expression and identity is performed independently, and processed in separate subsystems (Bruce and Young, 1986). However, during social interaction, it is important to be able to evaluate the differing changes in the emotional expressions of specific individuals engaged in the interaction (Baseler et al., 2012) and thus link identity and expression together. Indeed various lines of evidence have suggested an interaction between the processing of facial expression and face identity (see Calder and Young, 2005).

For example, single unit recordings in the monkey have shown that a proportion of neurons in the temporal cortex (Perrett et al., 1984; Hasselmo et al., 1989) and within the amygdala (Gothard et al., 2007) respond selectively to the conjunction of specific facial expressions and specific facial identities. Furthermore, psychophysical evidence (Schweinberger and Rudiger, 1998; Fox and Barton, 2007; Ellamil et al., 2008; Campbell and Burke, 2009) has supported the notion of a partially integrated processing of emotion and identity information. In addition, recent neuroimaging (e.g., Gobbini and Haxby, 2007; Vuilleumier and Pourtois, 2007; Baseler et al., 2012) and neuropsychological studies (e.g., de Gelder et al., 2003) have indicated that processing of facial emotion and facial identity can interact; also see Calder and Young (2005) for a discussion.

In a similar vein, voice identity and voice emotion have been proposed to be processed by separate, functionally independent systems (Belin et al., 2004; Spreckelmeyer et al., 2009). As for faces, these systems are not wholly independent, and the interactions between them may allow for voice emotion being processed with reference to the identity of the speaker (Belin et al., 2004).

In contrast, our understanding of how we conjointly process information about emotion and identity derived from body actions is still unclear and models of visual processing of actions (e.g., Giese and Poggio, 2003; Fleischer et al., 2013) have not been developed to take into account these factors. Therefore, in the current study, we investigated if identity played a role in the perception of emotional actions using visual adaptation paradigms. Visual adaptation results from prolonged exposure to a specific visual stimulus causing an aftereffect consisting of a bias in perception. Typically, perception of a test stimulus is biased toward the opposite of the adapted stimulus. Examining the characteristics of the aftereffects allows us to determine the properties of the underlying visual processing mechanisms, as has been used previously to understand the representation of other complex social stimuli like faces (e.g., Leopold et al., 2001; Webster et al., 2004; Fox and Barton, 2007), biological motion (e.g., Troje et al., 2006; Roether et al., 2009), implied motion (Lorteije et al., 2007), and actions (Barraclough et al., 2009; Barraclough and Jellema, 2011).

In our experiments, participants adapted to whole-body actions performed in either a happy or sad manner and then subsequently categorized test actions that were carried out in a neutral manner. Both physical characteristics of the body movement such as speed, acceleration or force, and more subtle relationships between the position of the joints inform about the type of expressed emotion and thus distinguish movements performed in happy or sad manner (e.g., Dittrich et al., 1996; Pollick et al., 2001, 2003; Roether et al., 2009). We chose happy and sad emotions for our adapting stimuli as they are opposite in valence with distinctive emotion-specific features (see Roether et al., 2009 for details). We predicted that emotional action adaptation would result in subsequent test actions to appear opposite in emotion to the emotion conveyed by the adapting action. We tested the role of identity in the emotional action aftereffects by varying the identity of the adapting and test actors. Following previous research (Kohn, 2007; Verhoef et al., 2008), we predicted that the magnitude of the aftereffect would depend upon the degree of similarity between the adapting and test stimuli. Additionally, in order to differentiate any putative emotional action aftereffect from other potential mechanisms, we performed three control experiments. First, to rule out the potential contribution of post-perceptual response and decision biases (cf. Hsu and Young, 2004) to the emotional action aftereffects we employed a 4AFC task in the adaptation experiment. Here we tested whether participants categorized test actions as conveying the opposite (cf. Leopold et al., 2001; Skinner and Benton, 2010), rather than a different emotion to that of the adapting action. Second, as adaptation aftereffects show characteristic build-up and decay over time (e.g., Hershenson, 1989; Leopold et al., 2005; Barraclough et al., 2012, but see Storrs, 2015), we examined these factors in two further experiments.

## General method

### Participants

Participants were University of Hull staff and students and received course credit or payment for participating. Participants had normal or corrected to normal vision. Number of participants tested in each experiment was based upon previously reported sample size in action adaptation studies (Experiment 1; c.f. Barraclough and Jellema, 2011; Barraclough et al., 2012) and determined by the power analysis on data obtained in pilot studies (Experiments 2–4). All participants were naïve to the purpose of the study except authors JI (Experiment 1) and JW (Experiments 2 and 4). Excluding authors' data from the analysis did not influence the significance of any results and thus any conclusions drawn. Experiments were approved by the ethics committee of the Department of Psychology, University of Hull, and performed in accordance with the ethical standards laid down in the 1990 Declaration of Helsinki.

### Stimuli

We created two sets of emotional action stimuli by filming actors performing different actions whilst conveying happy and sad emotions in varying degrees of intensity, as well as the same actions conveyed with no emotion (neutral). Emotion was conveyed by the way the actions were executed rather than by executing specific emblematic actions (e.g., see Atkinson et al., 2004; Keefe et al., 2014). Actors were free to interpret and express each emotion as they wished. We did not want to instruct actors to move in a specific way to ensure that a full range of naturalistic bodily expressions were represented in our stimulus sets. Furthermore, by allowing non-stereotyped bodily expressions of emotion from our actors, it was possible to maintain the unique kinematics of actions that help characterize the identity of the individual actors.

#### Stimulus set 1

Sixteen (eight females, eight males) lay actors with no specific acting experience were filmed. All actors were Caucasian and were students at the University of Hull. We recruited lay actors as previous studies (e.g., Roether et al., 2009; Keefe et al., 2014) show emotions portrayed by professional and lay actors are not significantly different from each other; lay actors portray emotional expressions that are easily recognized and similar to expressions of professional actors (e.g., Wallbott, 1998; Atkinson et al., 2007; Grèzes et al., 2007). Actors were filmed (Canon XL1s, 720 × 576 pixels, 25 fps, progressive scan) performing four different actions: lifting a box, putting down a box, sitting down on a chair, and walking to the right. Actors were asked to perform these actions in an emotionally neutral manner and whilst conveying happy and sad emotions in two levels of increasing intensity: moderate (somewhat happy/sad) and high (very happy/sad). Actors wore plain clothes (e.g., jeans and a dark t-shirt or jumper) and were filmed with their head at a three-quarter rear view (the face turned away from the camera), so that facial expressions could not be seen. This was done to ensure that the emotional expression and actor's identity are both conveyed only by bodily cues (shape and action kinematics) and not by the clothing or by the face. Recordings were edited to generate 320 (16 actors × 4 actions × 2 emotions × 2 intensity levels, plus neutral) 2 s long movies. These movies were subsequently rated by 14 independent observers (11 females, 3 males; mean age *M* = 21.9 years, *SD* = 4.58 years) on a five-point Likert scale, where 5 = very happy and 1 = very sad. The 64 actions (16 actors × 4 actions) rated as being most happy (*M* = 4.27, *SD* = 0.50), and the 64 actions (16 actors × 4 actions) rated as being most sad (*M* = 1.65, *SD* = 0.46) were used as adapting stimuli. The 64 neutral actions (16 actors × 4 actions; *M* = 2.88, *SD* = 0.28) were used as test stimuli.

#### Stimulus set 2

Stimuli in set 2 were selected from the Action Database (Keefe et al., 2014; http://www-users.york.ac.uk/~neb506/databases.html). This database contains movies of semi-professional actors with moderate acting experience (i.e., drama students at the University of York) and lay actors with no previous acting experience (i.e., non-drama students and staff at the University of York). As the lay actors were as able to portray different emotions through their bodily action similarly to the semi-professional actors (see Keefe et al., 2014 for details), for this study we selected sixteen (eight females and eight males) Caucasian actors. The full details of the filming and validation procedure for these stimuli are described in Keefe et al. (2014). Briefly, actors were filmed (Panasonic, HDC-TM900, HD format 1920 × 1080 pixels, at 50 fps progressive scan) in a chroma-green room walking diagonally to the right so that facial expressions could not be seen. They portrayed this action in an emotionally neutral manner whilst conveying emotions in three levels of increasing intensity: low (slightly happy/sad), moderate (somewhat happy/sad), and high (very happy/sad). All actors wore a gray t-shirt and dark trousers to ensure that actor's identity was conveyed by the bodily cues (shape and action kinematics) and not by the clothing. For this stimulus set, a mood induction procedure consisting of an autobiographical recall was used to help actors convey specific emotion during filming, and actors were free to convey the emotion as they wished. Film was edited to key out the green screen, and generate 112 (16 actors × 2 emotions × 3 intensity levels, plus neutral) 2 s long movies. Movies were subsequently rated by 10 (6 females, 4 males; mean age *M* = 27.1 years, *SD* = 9.6 years) independent observers on two independent nine-point Likert scales, where 1 = neutral, 9 = very happy/very sad, to ensure that the adapting actions conveyed the appropriate emotion (for detials of this rating procedure see Keefe et al., 2014).

For our purposes we chose the 16 actors from the database that conveyed the happy and sad walking actions with the highest intensity. For each actor, actions rated as happy (16 movies, *M* = 5.31, *SD* = 1.34) and sad (16 movies, *M* = 4.56, *SD* = 1.02) were selected as adapting stimuli. Neutral actions (16 movies, also rated for happiness: *M* = 2.77, *SD* = 0.47, as well as sadness: *M* = 2.89, *SD* = 0.56) were used as test stimuli.

### Procedure

**Figure 1:**
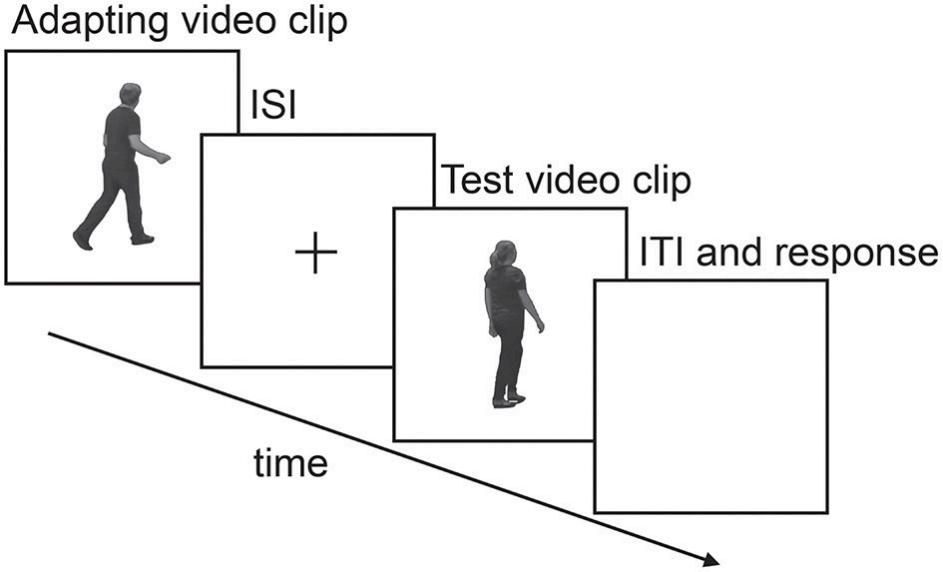
**A schematic illustration of a typical single experimental trial**. Duration of the adapting stimulus, inter-stimulus interval (ISI) and test stimulus differed across experiments and is specified in each method section for each experiment.

A PC running MATLAB 2006a (The MathWorks Inc., Natick, MA) and the Cogent toolbox (www.vislab.ucl.ac.uk/cogent.php) was used to control the experiment, display movies in the center of a 22” CRT monitor (Philips 202P40, 1600 × 1200 pixels, 100 Hz refresh rate), and record participant responses. Participants sat ~57cm from the screen. Movies from stimulus set 1 were presented such that they filled the entire screen and subtended ~41.3 degrees × 31.5 degrees at the eye. Movies from stimulus set 2 were presented on a black background such that the full width of the HD movie fitted the horizontal extent of the screen. To help preclude low-level retinotopic dependent adaptation, participants freely viewed all stimuli limiting stereotypic eye-movements. All experiments followed a standard adaptation procedure (Figure 1) where adapting movies were followed by a blank inter-stimulus interval (ISI) containing a small yellow fixation cross, followed by a single test movie. Participants were required to indicate on a labeled keypad whether the test movie conveyed happy or sad emotions (2 AFC: Experiments 1, 3, and 4) or happy, sad, disgusted or surprised emotions (4 AFC: Experiment 2).

### Data analysis

For each experiment and each participant and condition, we calculated the mean aftereffect (see individual sections). Even though aftereffects with complex social stimuli are typically quite variable (Barraclough and Jellema, 2011; Barraclough et al., 2012) a very small number of participants showed exceptionally large aftereffects. These effects may reflect a potential problem in the conduct of the task by these individuals (for example rating the adapting stimulus, and not the test stimulus). Data was therefore discarded from those outlying individuals with aftereffects more than 2 SD from the mean aftereffect. Whenever data from outlying participants have been excluded, and whether its exclusion influenced the significance of the results, has been described in the respective results section.

## Experiment 1: adaptation to emotional actions

Experiment 1 tested if exposure to movies of actors portraying happy and sad emotions through their bodily actions resulted in emotional action aftereffects: a change in the perception of neutral actions biased toward the opposite of the adapted emotion. The adaptation paradigm was adapted from Fox and Barton (2007), who investigated the role of identity in emotion perception from faces. The effect of stimulus similarity on the magnitude of emotional action aftereffects was tested by varying the similarity between actions executed in the adapting and test stimuli, and by varying the similarity between the identities of the actors executing the actions.

### Method

#### Participants

Twenty participants (14 females, 6 males, mean age = 23.85 years, *SD* = 8.2 years) took part in the experiment.

#### Stimuli

Stimuli were selected from stimulus set 1 (see General method section): two females and two males actors performing three actions (lifting a box, sitting down, and walking). Actions performed in a happy and sad manner were used as adapting stimuli, while actions performed in a neutral manner were used as test stimuli.

#### Procedure

Stimuli were presented across five blocks, each containing 24 trials. In an initial control block without adaptation, 12 test stimuli (depicting four actors performing three actions in a neutral manner) were presented twice in a pseudorandom order. Participants categorized the emotion conveyed by the actor as either happy or sad (scored as: 1 = happy, 0 = sad). The control block was later used as a baseline for calculating the aftereffects. The control block was followed by four counterbalanced adaptation blocks, where 4 different pairings of adapting and test stimuli were employed, where the adapting and test stimuli had the same identity/same action, same identity/different action, different identity/same action, or different identity/different action. Adapting stimuli (happy and sad actions) were presented for 16 s (each movie was repeated eight times), followed by a 0.3 s ISI, and then a test stimulus presented for 2 s. Each test stimulus was presented following both happy and sad adaptation stimuli, where this factor was interleaved within the block. Participants categorized the emotion conveyed by the test actor as either happy or sad (scored as: 1 = happy, 0 = sad). Following the participant response there was an inter-trial interval (ITI) of 5 s before the start of the next trial. Mean estimates of the emotion perceived in the test stimuli were calculated separately for the control, and happy and sad adaptation conditions across the 4 stimulus similarity conditions. “Happy” and “sad” aftereffects were calculated by subtracting estimates of test stimuli in the control condition from the happy and sad adaptation conditions, respectively.

#### Results

**Figure 2:**
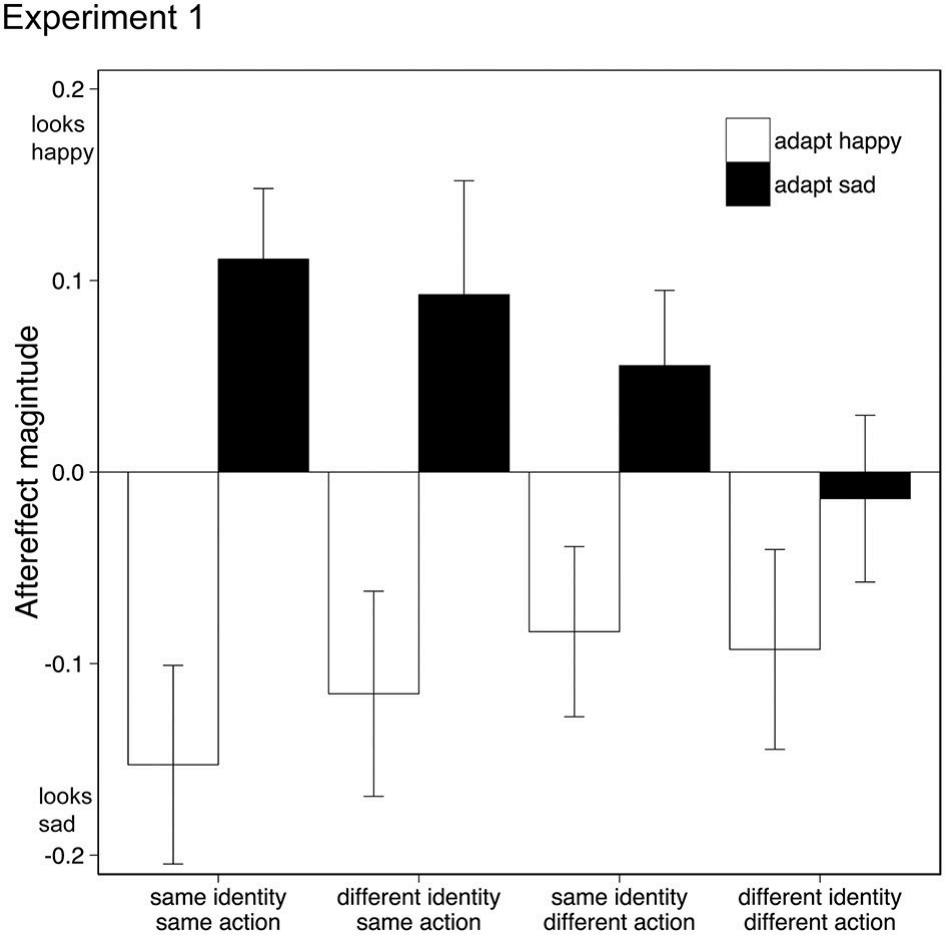
**Emotional action aftereffects for different pairings of adapting and test actions**. Actions executed by the adapting and test actors could either be the same or different (same action, different action), and the identity of the actor in the adapting and test stimuli could either be the same or different (same identity, different identity). Positive values indicate that adaptation made the test actors appear happier (than in the no-adaptation condition), negative values indicate that adaptation made the test actors appear sadder. Error bars represent standard error of the mean (SEM).

Data from two participants (both female, both age 20) were discarded from the analysis as one showed outlying data (>2 SD from mean aftereffect) and one did not engage in the task. Removal of this data did not change the significance of the following analysis. Aftereffects were tested using a within-subject Analysis of Variance (ANOVA) with factors adaptation emotion (happy, sad) and stimulus similarity (same identity/same action, same identity/different action, different identity/same action, different identity/different action). Adaptation to happy and sad emotional action movies generated significantly different aftereffects {*F*_(1, 17)_ = 11.261, *p* < 0.005, η_*p*_^2^ = 0.40, 95% CIs: adapt happy [−0.21, −0.015], adapt sad [−0.01, 0.14], see Figure 2}, where test actions were judged as conveying the opposite emotion to the adapting stimuli, similar to other emotional aftereffects seen with biological motion stimuli (Roether et al., 2009) and facial expressions (Fox and Barton, 2007). As the similarity between the adapting and test stimuli decreased, so did the aftereffect magnitude {interaction between adaptation emotion and stimulus similarity: *F*_(2.1, 35.7)_ = 4.25, *p* < 0.05, η_*p*_^2^ = 0.20, 95% CIs adapt happy [−0.26, −0.04], [−0.18, 0.01], [−0.23, 0.0], [−0.20, 0.02], adapt sad [0.03, 0.19], [−0.03, 0.14], [−0.03, 0.22], [−0.11, 0.08] for each level of similarity respectively, Greenhouse-Geisser correction applied}; there was no main effect of stimulus similarity {*F*_(2.1, 35.8)_ = 0.83, *p* > 0.05, η_*p*_^2^ = 0.05, 95% CIs [−0.09, 0.05], [−0.08, 0.05], [−0.10, 0.08], [−0.13, 0.03], Greenhouse-Geisser correction applied}.

Happy and sad aftereffects were significantly different only in those conditions where the adapting and test stimuli contained the same action, irrespective of identity: same identity/same action {*t*_(17)_ = 4.48, *p* < 0.001, 95% CIs [0.14, 0.39]}; different identity/same action {*t*_(17)_ = 2.85, *p* = 0.011, 95% CIs [0.05, 0.36], Bonferroni correction applied}. For conditions where the adapting and test stimuli contained different actions, happy and sad aftereffects were not significantly different from each other, indicating that adaptation to emotional action adaptation does not transfer from one action type to another.

## Experiment 2: control for change in response criterion

In Experiment 2 we clarified whether the emotional action aftereffects were due to biases in perception rather than post-perceptual processes, for example biases in participant responses or decisions. We reasoned that aftereffects resulting from a perceptual process would result in test actions being categorized as conveying the opposite emotion following adaptation (see Hsu and Young, 2004; Webster et al., 2004). Here, perceptual adaptation to one emotion (e.g., happy) would result in an increase in the likelihood of only the opposite emotion (sad), rather than other possible emotions. If, however, the aftereffects resulted from either a bias in response or decision then participants would be likely to indicate that the test actions conveyed “any other” emotion to that of the adapting stimulus. In these cases, participants could in principle have reasoned that since the test stimulus was clearly different from the adapting stimulus, they should select a response option that was clearly different from the adapting stimulus.

We tested these predictions across the two conditions that generated significant aftereffects in Experiment 1 (same action/same identity and same action/different identity). Further, to check that these two emotional action aftereffects were not dependent upon any particular set of stimuli, we selected movies from stimulus set 2 (see General method).

### Method

#### Participants

Fifteen new participants (six females, nine males, mean age = 24.9 years, *SD* = 3.2 years) took part in the experiment, all were naïve to the purpose of the experiment except author JW, and one individual (male, age 28) tested also during Experiment 4. Removal of these data did not change the significance of the following analysis.

#### Stimuli

Stimuli were obtained from stimulus set 2 (see General method). Eight females and eight males actors performing walking actions were selected for the experiment. Walking actions performed in a happy and sad manner were used as adapting stimuli, while walking actions in a neutral manner were used as test stimuli.

#### Procedure

Stimuli were presented across four blocks; an initial control block without adaptation (the 16 test stimuli presented twice in a pseudorandom order), followed by two counterbalanced adaptation blocks (where on each trial adapting and test stimuli were paired to create same identity/same action and different identity/same action combinations of stimulus similarity), and a final control block identical to block 1. The control condition was split across two blocks (before and after adaptation), in order to obtain a stable measure of test stimulus emotion over the duration of the experiment. Adapting stimuli (happy and sad actions) were displayed for 8 s (by repeating the movie four times); the ISI was 0.4 s. Each test stimulus was presented following happy and sad adaptation. Participants categorized each test movie as happy, sad, surprised or disgusted (a 4AFC task). Surprise and disgust were selected as additional options as they vary from happiness and sadness in valence and arousal (Russell, 1980). Following the participant response there was an inter-trial interval of 5 s before the start of the next trial. For each participant, and each adaptation block, we calculated percentage of responses for each of the 4 possible emotions (happy, sad, surprised, and disgusted; cf. Skinner and Benton, 2010). We averaged the responses from both control blocks and calculated the percentage of each response as for the adaptation blocks. Aftereffects were calculated by subtracting the percentage of each response from the control blocks from percentage of each response to the same stimuli following happy and sad adaptation for same and different identity conditions separately.

#### Results

**Figure 3:**
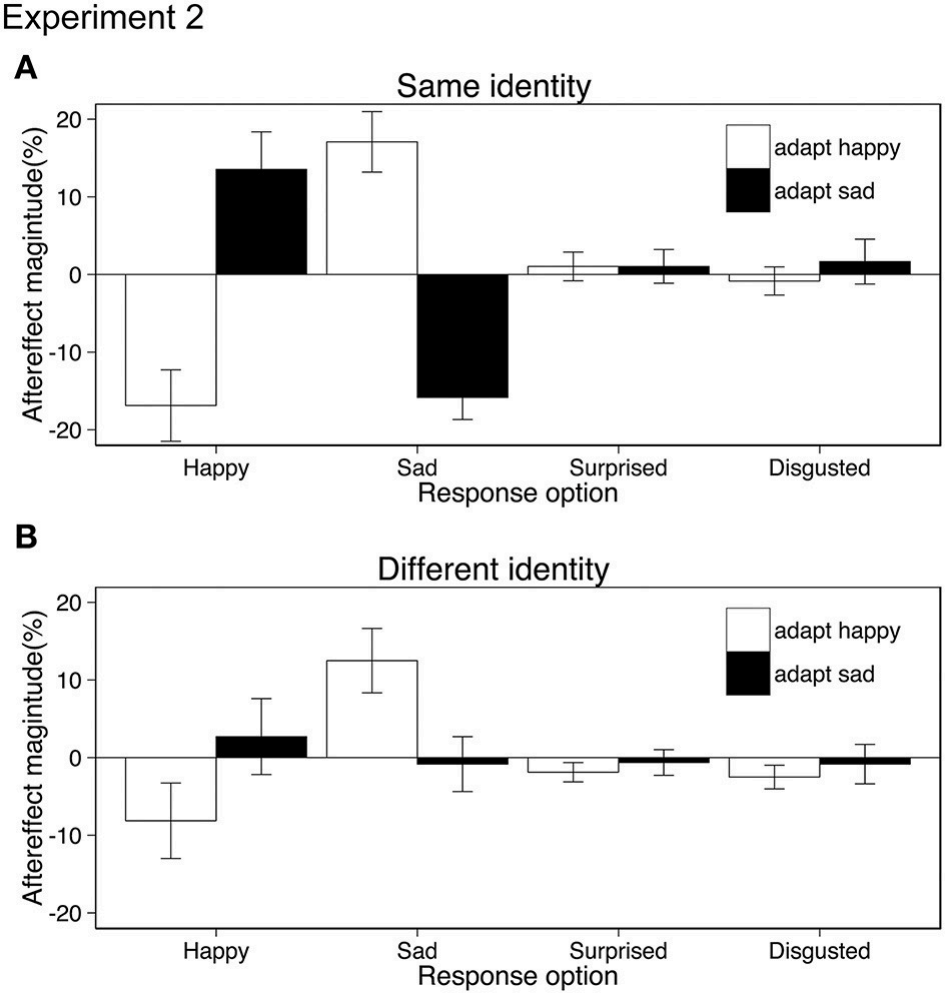
**Aftereffect magnitude calculated as the change in the mean percentage of the responses for each of the four possible emotions conveyed by test stimuli following happy and sad adaptation (positive values indicate more often selected emotion following adaptation; negative values indicate less often selected emotion following adaptation)**. The identity of the actors in the adapting and test stimuli were either the same **(A)** or different **(B)**. Error bars represent standard error of the mean (SEM).

Aftereffects were tested using ANOVA with the within-subject factors of adaptation emotion (happy, sad), stimulus similarity (same identity/same action, different identity/same action) and response emotion (happy, sad, surprised, disgusted). Both the same and different identity aftereffects resulted from shifts in the perception of action emotion (rather than a bias in response or decision) as participants categorized test movies as conveying the opposite to the adapted emotion rather than “any other” emotion {interaction adaptation emotion × response emotion, *F*_(1.81, 25.4)_ = 27.20, *p* < 0.001, η_*p*_^2^ = 0.66; 95% CIs adapt happy [−22.05, −7.00], [7.01, 22.51], [−3.54, 2.71], [−4.72, 1.39], adapt sad [−1.07, 17.32], [−14.59, −2.07], [−2.82, 3.23], [−4.34, 5.17] for happy, sad, surprised, disgusted response option respectively, Greenhouse-Geisser correction applied, Figure 3}. As suggested by Figure 3, the same identity aftereffects were significantly bigger (*M* = 15.8) than the different identity aftereffects (*M* = 6.0). This was confirmed by a three-way interaction (adaptation emotion × stimulus similarity × response emotion, ANOVA, *F*_(3, 42)_ = 14.49, *p* < 0.001, η_*p*_^2^ = 0.50, see Appendix for 95% CIs). No other main effects or interactions reached significance. *Post-hoc* paired *t*-tests revealed that adaptation only affected the categorization of test stimuli as happy {same identity: *t*_(14)_ = 5.88, 95% CIs [−41.51, −19.32] different identity: *t*_(14)_ = 3.11, 95% CIs [23.17, 42.66], both *ps* < 0.0125, Bonferroni correction applied}, and sad {same identity: *t*_(14)_ = 7.25, 95% CIs [−18.28, −3.37], different identity: *t*_(14)_ = 3.38, 95% CIs [4.87, 21.78], both *ps* < 0.0125, Bonferroni correction applied} and had no effect on the choice of surprised or disgusted labels (all *ps* > 0.27).

## Experiment 3: dynamics of emotional action aftereffects

The first two experiments indicated that adaptation influences perceptual mechanisms that represent emotional actions, and that these mechanisms are selective for the executed action. We find that when the identities of the adapting and test actors are the same, aftereffects are greater than when the identities of the adapting and test actors are different (Figures 2, 3). Similar to the face aftereffects observed by Fox and Barton (2007) this indicates that action emotion can be represented irrespective of actor identity, but also actor identity can influence the representation of action emotion. These results suggest that there are two potential mechanisms underlying the coding of action emotion that are both susceptable to percetual adaptation. Adaptation aftereffects seen with other stimuli show a characteristic logarithmic build-up and exponential decay over time, e.g., for motion (Hershenson, 1989), faces (Leopold et al., 2005; Rhodes et al., 2007), biological motion (Troje et al., 2006), and actions (e.g., Barraclough et al., 2009). To further test whether our two emotional action aftereffects show the same characteristic dynamics as previously reported perceptual aftereffects we examined their build-up and decay over time. We tested the effect of adapting stimulus duration and the duration of the inter-stimulus interval (ISI) between the adapting and test stimuli on aftereffect magnitude in two separate, fully factorial experiments (Experiment 3a: same identity/same action aftereffect; Experiment 3b: different identity/same action aftereffect). Furthermore, these results would help further distinguish the perceptual aftereffects we observe from other post-perceptual processes.

### Method

#### Participants

Nineteen new participants (11 females, 8 males, mean age = 20.4 years, *SD* = 3.8 years) took part in Experiment 3a; a further 18 new participants (12 females, 6 males, mean age = 24.7 years, *SD* = 6.7 years) took part in Experiment 3b. All participants were naive to the purpose of the experiment.

#### Stimuli

Stimuli were obtained from stimulus set 1 (see General method). Eight females and eight males actors performing four different actions (lifting a box, putting down a box, sitting down on a chair, and walking to the right) were selected for the experiment. Actions performed in a happy and sad manner were used as adapting stimuli, while actions in a neutral manner were used as test stimuli.

#### Procedure

In both experiments we varied the duration of the adapting stimulus (2, 4, 8, 16 s) by repeating the adapting movie a number of times (1, 2, 4, 8 repeats), and varied the ISI between the adapting and test stimuli (200, 400, 600, 1600 ms in Experiment 3a; 400, 800, 1600, 3200 ms in Experiment 3b). In each experiment, therefore, there were 32 different adaptation conditions (4 adaptation repeats × 4 ISIs x 2 emotions) that occurred in a pseudorandom order. The choice of adaptation durations and ISIs in Experiment 3a was based upon previous measures of the duration of face identity aftereffects (Leopold et al., 2005) and whole body aftereffects (Barraclough et al., 2012) where significant aftereffect declines were observed over 1600 ms. Following Experiment 3a we conducted Experiment 3b, here we decided to extend the range of tested ISIs to 3200 ms to better sample the possible decline of aftereffects over time. In Experiment 3a the identity of the actor and the type of action performed in the adapting and test movies were identical on every trial. In Experiment 3b, the type of action performed by the actor in the adapting and test movies was identical on every trial, however, the identity (and gender) of the actor was always different. Participants completed 320 trials in total over two testing sessions. Participants categorized the emotion conveyed by the test actor as either happy or sad (scored as: 1 = happy, 0 = sad). Following the participant response there was an inter-trial interval (ITI) of 5 s before the start of the next trial.

Aftereffects were calculated by subtracting the mean ratings of the test stimuli following adaptation to sad stimuli from the mean ratings of the test stimuli following adaptation to happy stimuli. This difference value represented the magnitude of the aftereffect. Here, positive values indicated typical, repulsive aftereffects, where following adaptation the emotion conveyed by the test actors looked like the opposite of the emotion conveyed by the adapting actors. Negative aftereffects values indicated that following adaptation, the emotion conveyed by the test actors looks similar to the emotion conveyed by the adapting actors. Although this method precludes identifying the relative contribution of happy and sad actions to emotional aftereffect dynamics, it reduces any systematic biases, maximizes the magnitude of any aftereffects and eliminates the necessity for a control condition without adaptation, thereby allowing us to test many experimental conditions within a limited testing period.

#### Results

**Figure 4:**
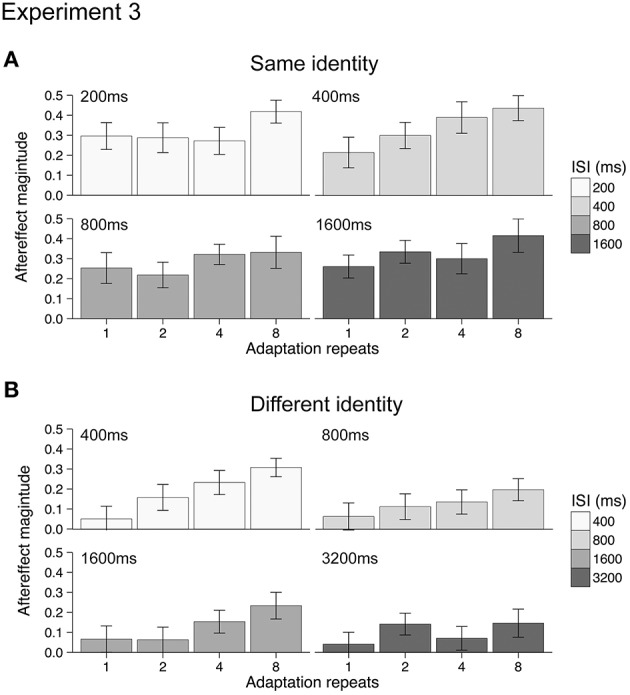
**Emotional action aftereffects magnitude as a function of adaptation repeats and ISI duration for Experiment 3a (A) and Experiment 3b (B)**. All aftereffects showed positive values typical of repulsive aftereffects, where test stimuli looked more like the opposite of the adapting stimuli. Error bars indicate SEM.

Mean aftereffects for the 16 different conditions were calculated in both Experiments 3a and 3b and are plotted in Figure 4. Data from one participant (female, age 20) in Experiment 3a was discarded from the analysis as outlying data. Repeating the analysis with this data did not change the significance of the results when testing with ANOVA. For both Experiments 3a and 3b, all aftereffects were positive, indicating that under all conditions adaptation had a repulsive effect. The dynamics of the emotional action aftereffects, however, appeared to be different in the two experiments (compare Figures 4A,B). As the groups of participants and the durations of the ISIs between the two experiments were different, we analyzed the two experiments separately using 2-way ANOVAs (4 adaptation repeats × 4 ISI durations).

For Experiment 3a (same identity) increasing the duration of the adapting stimulus results in significantly larger aftereffects [main effect of adaptation repeats: *F*_(3, 51)_ = 5.72, *p* < 0.005, η_*p*_^2^ = 0.25, see Appendix for 95% CIs]. There was, however, no significant effect of ISI duration [main effect of ISI: *F*_(3, 51)_ = 1.04, *p* = 0.38, η_*p*_^2^ = 0.06, see Appendix for 95% CIs] nor a significant interaction between adaptation repeats and ISI duration [adaptation repeats × ISI: *F*_(9, 153)_ = 0.90, *p* = 0.53, η_*p*_^2^ = 0.05, see Appendix for 95% CIs]. For Experiment 3b (different identity) increasing the duration of the adapting stimulus results in significantly larger aftereffects [main effect of adaptation repeats: *F*_(3, 51)_ = 9.94, *p* < 0.001, η_*p*_^2^ = 0.37, see Appendix for 95% CIs]. Although a reduction in aftereffect magnitude with ISI was apparent from Figure 3B (i.e., overall reduction in the size of bars across ISI durations), this was not significant [main effect of ISI: *F*_(3, 51)_ = 1.95, *p* = 0.132, η_*p*_^2^ = 0.10, see Appendix for 95% CIs] nor was the interaction between adaptation repeats and ISI significant [adaptation repeats × ISI: *F*_(9, 153)_ = 0.90, *p* = 0.52, η_*p*_^2^ = 0.05, see Appendix for 95% CIs].

The reason for the lack of significance of ISI on aftereffect magnitude in Experiment 3b may be due to a number of factors. First, following 1 repeat of the adapting stimulus there was little aftereffect at any ISI. Indeed, significant aftereffects were only found following 4 or 8 adaptation stimulus repeats (one-sample *t*-tests, *p* < 0.0031, Bonferroni correction applied). This contrasts with the analysis of the same identity aftereffects measured in Experiment 3a, where significant aftereffects were found following 1, 2, 4, and 8 adaptation stimulus repeats (one-sample *t*-tests, *p* < 0.0031, Bonferroni correction applied). The small and non-significant aftereffects in Experiment 3b obtained after 1 and 2 repeats may have contributed to the non-significant main effect of ISI. Second, the range of ISIs that we tested for both Experiments 3a and 3b may have been too restricted, with aftereffects tested over a greater range of ISIs we may have found a significant decline in the magnitude of both same identity and different identity aftereffects.

**Figure 5:**
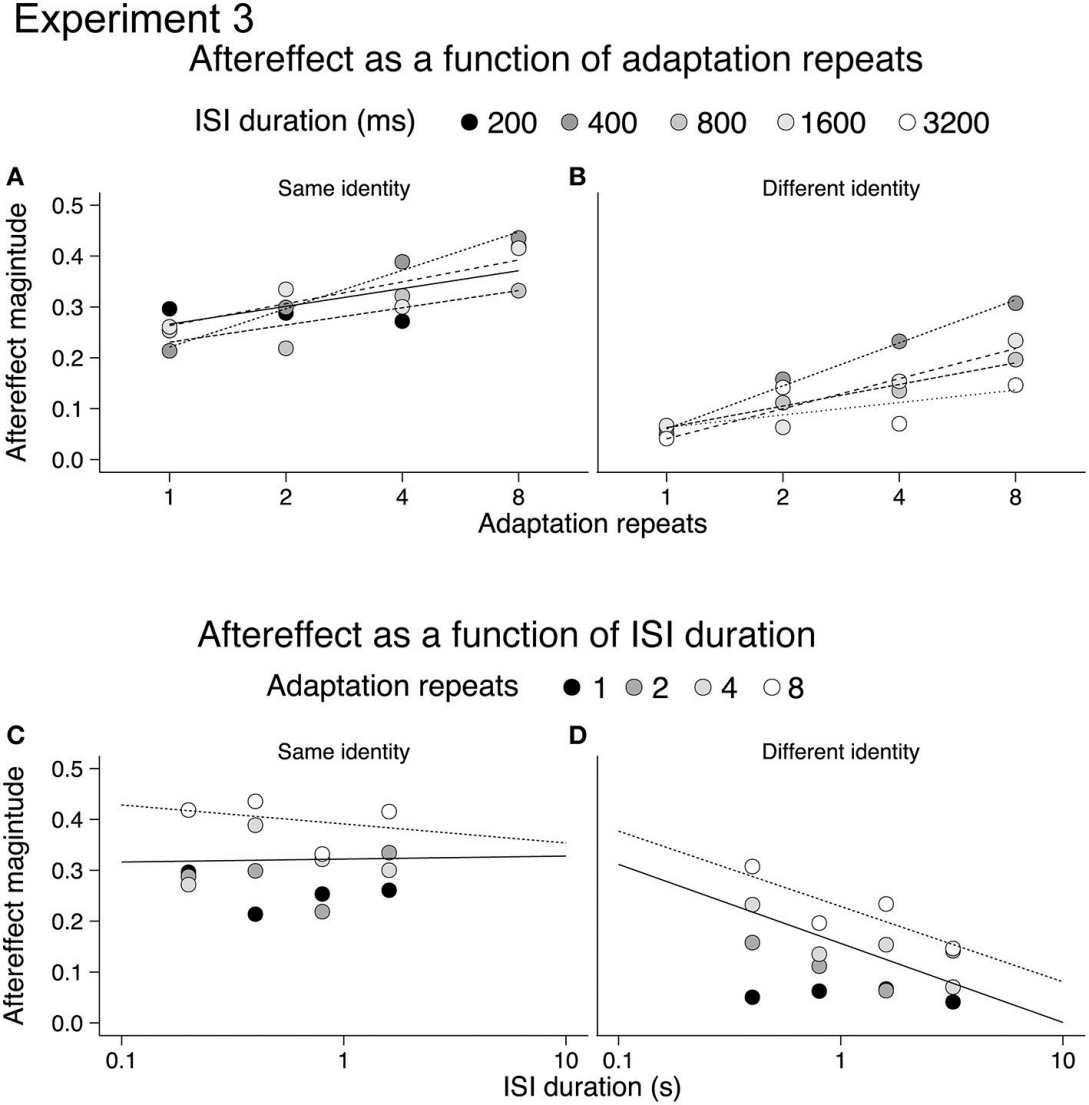
**Same identity (A,C) and different identity (B,D) aftereffects plotted on a semi-log scale**. Aftereffects build-up with adaptation repeats **(A,B)**, and aftereffects decline with ISI **(C,D)**.

In order to better illustrate the build-up and decay of the emotional action aftereffects we re-plotted the aftereffects for same and different identity conditions on a semi-log scale, as a function of adaptation repeats (Figures 5A,B) and ISI duration (Figures 5C,D; cf. Leopold et al., 2005). The lack of decay of the aftereffects in the same identity condition raised a possibility that same identity and different identity aftereffects show a difference in storage. Storage occurs when a period of non-stimulation (e.g., darkness) is imposed between the adapting and the test stimulus. In consequence, the decay of an aftereffect is delayed as it persists for a period of non-stimulation (Thompson and Wright, 1994). Storage has previously been reported for motion aftereffects (e.g., Thompson and Wright, 1994; Watamaniuk and Heinen, 2007) and face aftereffects (Leopold et al., 2001; Hsu and Young, 2004) suggesting that emotional action aftereffects may also exhibit a storage effect. Therefore in Experiment 4 we tested the decay of emotional action aftereffect over a short (400 ms) and long (10.8 s) ISI durations.

## Experiment 4: aftereffect duration tested with longer inter-stimulus intervals

In Experiment 4 we wanted to assess whether the lack of significant decline with time observed in Experiments 3a and b was a function of the ISIs originally chosen, and whether the similarity of the identity of the actor in the adapting and test stimuli had an influence on the duration of aftereffects. Experiments 3a and 3b gave an indication for the duration of emotional action aftereffects following 4 repeats of adapting stimuli. At short ISIs (400 ms) we expected to see significant aftereffects in the same identity/same action condition, as well as in the different identity/same action condition. At long ISIs (10.8 s), however, we expected to see no decline in the same identity/same action condition, but a decline in the different identity/same action condition.

### Methods

#### Participants

Twenty-four new participants (16 females, 8 males, mean age = 21.5 years, *SD* = 3.1 years) took part in the experiment; all were naïve to the purpose of the experiment except author JW.

#### Stimuli

Stimuli were obtained from stimulus set 1 (see General methods). Four females and 4 males actors performing four different actions (lifting a box, putting down a box, sitting down, and walking) were selected for the experiment. Actions performed in a happy and sad manner were used as adapting stimuli, while actions performed in a neutral manner were used as test stimuli.

#### Procedure

Stimuli were presented across two counterbalanced blocks, where on each trial adapting and test stimuli were paired up to create same identity/same action or different identity/same action stimulus similarity conditions. Adapting stimuli (happy or sad actions) were presented for 8 s (repeated four times). Inter-stimulus intervals, determined in pilot testing, were 400 ms and 10.8 s. Each test stimulus was presented following happy and sad adaptation and each ISI. Participants categorized the emotion conveyed by the test actor as either happy or sad (scored as: 1 = happy, 0 = sad). Following the participant response there was an inter-trial interval (ITI) of 5 s before the start of the next trial. We calculated mean estimates of test stimuli following happy and sad adaptation separately for each of the four different conditions (identity × ISI). We then calculated aftereffect magnitudes by subtracting responses following sad adaptation from responses following happy adaptation.

#### Results

**Figure 6:**
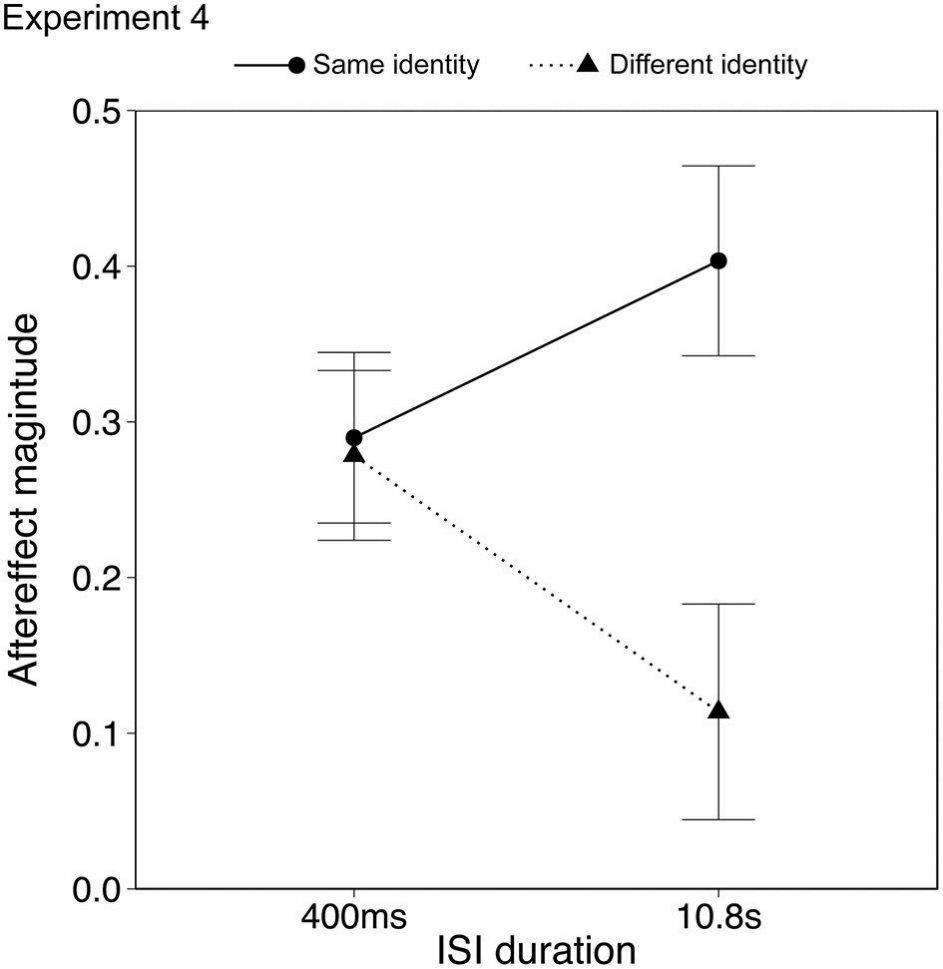
**Emotional action aftereffects as a function of the ISI**. Error bars represent standard error of the mean (SEM).

Data from two participants (male, age 19; female, age 20) were discarded from the analysis as they showed outlying data (>2 SD from mean aftereffect). Results with and without the outlying data are described below. Aftereffects were analyzed using ANOVA with within-subject factors of identity (same, different) and ISI duration (0.4, 10.8 s). The aftereffects in the same identity/same action condition were significantly larger than the aftereffects in the different identity/same action condition, as seen in the previous two experiments {Figure 6; ANOVA, main effect of identity, *F*_(1, 21)_ = 5.74, *p* < 0.05, η_*p*_^2^ = 0.25, 95% CIs [0.20, 0.44], [0.09, 0.30] for same and different identity respectively}. Importantly there was a significant interaction between identity and ISI {*F*_(1, 21)_ = 4.77, *p* < 0.05, η_*p*_^2^ = 0.18, 95% CIs; same identity [0.16, 0.40], [0.21, 0.51]; different identity [0.13, 0.37], [−0.01,0.28] for 0.4 s and 10.8 s ISI respectively}, in which the aftereffect in the different identity/same action condition declined over time, whereas the aftereffect in the same identity/same action condition appeared to persist. There was no main effect of ISI {*F*_(1, 21)_ = 0.07, *p* = 0.79, η_*p*_^2^ = 0.00, 95% CIs [0.16, 0.37], [0.14, 0.36] for same and different identity respectively}. Including the outlying data from the two participants who showed strong priming effects influenced only the identity × ISI interaction which became a trend {*F*_(1, 23)_ = 3.66, *p* = 0.06, η_*p*_^2^ = 0.14, 95% CIs; same identity [0.07, 0.36], [0.14, 0.46]; different identity [0.06, 0.33], [−0.05, 0.25] for 0.4 and 10.8 s ISI respectively}. The aftereffects in the different identity/same action condition therefore appeared to show a similar decline over time as for other high level and action aftereffects (Leopold et al., 2005; Troje et al., 2006; Barraclough et al., 2012). The aftereffects in the same identity/same action condition, however, showed a different pattern.

## Discussion

Here we show that adaptation to naturalistic movies of whole-body actions results in emotional aftereffects. Adaptation to an actor conveying either happiness or sadness biases subsequent perception of emotion conveyed by either the same or another individual to appear like the opposite emotion. Importantly, we see that the characteristics of the aftereffects are dependent upon whether the subsequent individual has the same or a different identity to the adapting actor.

The effects observed here are likely to result from adaptation of high level action coding mechanisms that represent the emotions of actors. The emotional action aftereffects show several of the characteristics of previously observed high-level aftereffects. First, similarity between adapting and test stimuli determined aftereffect magnitude (see Kohn, 2007; Verhoef et al., 2008). Aftereffects were largest when the identity and the action of the adapting and test stimuli were the same. As the identity or the action differed between these stimuli, aftereffects became smaller. In addition, aftereffects failed to transfer across different actions suggesting that the aftereffects did not result from cognitive biases, where decision regarding the test stimuli could be based purely on the valence information. Second, the adaptation aftereffects observed here did not result from a change in the response or decisions made by the participants during the experiments. Experiment 2 showed that participants did not alter their response following adaptation; rather, they selectively categorized the emotion of the test stimuli as the emotion opposite to the adaptor's emotion (discussed in Hsu and Young, 2004). Third, aftereffect magnitude increased with adaptation exposure as for other high-level action and face aftereffects (e.g., Leopold et al., 2005; Barraclough et al., 2012, but see Storrs, 2015). Fourth, the aftereffects that resulted from the different identity/same action condition appeared to decline over time, similar to other high-level action and face aftereffects (e.g., Leopold et al., 2005; Barraclough et al., 2012). The aftereffects that resulted from the same identity/same action condition, however, appeared to show a storage effect, similar to motion (e.g., Thompson and Wright, 1994; Watamaniuk and Heinen, 2007) and face aftereffects (Leopold et al., 2001; Hsu and Young, 2004).

Finally, the dominating effect here was the effect of high-level emotion adaptation, as the aftereffect was predominantly determined by the emotion conveyed by the adapting actor. Low-level retinotopic dependent adaptation is unlikely to explain the effects that we observe here, as adapting and test stimuli were dynamic with naturally occurring differences in expressions of emotions and kinematics across actors. Furthermore, actors were never presented in precisely overlapping retinotopic locations (comparable to e.g., Butler et al., 2008; Xu et al., 2008). Together, these results rule out other possible low-level or post-perceptual explanations for emotional action aftereffects that might account for some other demonstrations of high-level perceptual aftereffects.

We cannot, however, rule out the contribution of adaptation in motion processing mechanisms, which is likely to occur simultaneously when viewing complex action stimuli (e.g., Benton et al., 2016). Action speed is an important cue in the emotion recognition from body actions (e.g., Dittrich et al., 1996; Pollick et al., 2001, 2003; Roether et al., 2009), and is likely to have influenced the aftereffects observed here, but is unlikely to be a dominating factor. Other postural and dynamic features that do not rely on speed are also critical for expression and for perception of emotional actions (e.g., Atkinson et al., 2007; Roether et al., 2009). These postural (e.g., head angle) and dynamic (e.g., expressiveness) features present in happy and sad actions, even when these are matched for speed, are sufficient to elicit emotional action aftereffects (Roether et al., 2009). It is therefore plausible that the aftereffects observed here resulted from adaptation at multiple levels of action visual processing.

Importantly, our studies with emotional action aftereffects indicate that identity plays a role in the representations of emotional actions, and identity can modulate the way that emotional actions are processed. When the identities of the adapting and test actors were the same aftereffects were larger than when the identities of the adapting and test actors were different. Furthermore, when the identities of the adapting and test actors were the same, the aftereffects did not appear to decline over the time period we tested (up to 10.8 s). In contrast, when the identities of the adapting and test actors were different, the aftereffects declined over time. Thus, emotional action aftereffects show different magnitudes and decay functions dependent upon the relationship between the identity of adapting and test actors.

One explanation for our results is that emotional actions are represented in both an identity-dependent and identity-independent way. The aftereffects observed following adaptation in the different identity/same action condition might result from adaptation of a mechanism that represents emotional actions in an entirely identity-independent fashion. Whilst the aftereffects observed following adaptation in the same identity/same action condition might result from adaptation of mechanisms that represent emotional actions in both an identity-independent as well as an identity-dependent way. When adapting and test actors have the same identity, the aftereffects increase in magnitude and appear to last much longer than typical high-level action or face aftereffects. The increase in aftereffect magnitude could be explained by an increase in visual similarity between the adapting and test stimuli. However, this increase in visual similarity could not abolish the decay of the aftereffect over time in the way that we observe here. A parsimonious explanation is that an identity-dependent mechanism is additionally adapted when adapting and test stimuli have the same identities, resulting in a larger aftereffect and a longer-lasting influence on the representations of the emotions of individual actors. Such long-lasting aftereffects are not necessarily unusual, as they have been previously reported with other social stimuli such as gaze direction (e.g., Kloth and Schweinberger, 2008) and face configuration (e.g., McKone et al., 2005; Carbon and Ditye, 2011, 2012). These previous effects, however, were observed with much longer adaptation periods than we have used in our experiment reported here. Identity has also been shown to affect the decay of face aftereffects (Kiani et al., 2014). Brief presentation of an unrelated, intervening face in the period between the adaptation and the test phase, accelerated the decay of face aftereffects by 85%, whilst presentation of a face of an opposite to the adapted identity did not affect the decay (Kiani et al., 2014). However, it is not yet clear whether other inferences could have contributed to the quick decay of aftereffects in the different identity/same action condition observed here.

For both identity-dependent and identity-independent aftereffects, adaptation never transferred across different actions. Different actions have different kinematics, and bodily expression of emotion is dependent upon the characteristic kinematics of the action (e.g., see Roether et al., 2009). The failure of adaptation to one set of characteristic movements to exert an influence on the perception of a very different set of movements, suggests that actor emotion may be coded within action specific neural mechanisms.

In some ways, these results parallel findings of face emotion aftereffects. Evidence from face adaptation studies suggest the existence of a common visual representation that underlies the coding of face identity and face expression (Ellamil et al., 2008; Rhodes et al., 2015). This common representation may contain one dimension that codes both the identity and expression, and the other dimension that is selective for identity or expression (Calder, 2011; Rhodes et al., 2015). These findings are also consistent with Haxby et al.'s (2000) model of face perception, where neural substrates that underlie processing of invariant (e.g., identity) and changeable (e.g., expression) aspects of the face are thought to be disocciable, although the model also suggests they may interact. Specifically, the fusiform face area (FFA), predominantly involved in the coding of the invariant aspects of the face, may also play a supportive role in the coding of emotional expressions (Haxby et al., 2000).

Similarly, processing of voice identity and voice emotion is thought to be processed by seperate, but interacting systems (Belin et al., 2004; Spreckelmeyer et al., 2009), where voice identity is processed within the anterior STS (Belin et al., 2004) and voice emotion is processed by several separate regions including the amygdala, anterior insula, and temporo-medial and inferior prefrontal cortex (Imaizumi et al., 1997; Buchanan et al., 2000). While early analysis of vocal input (from primary auditory cortex to the middle part of the STS) is shared for different types of vocal information, at the highest level of voice processing the pathways become independent (Belin et al., 2004).

The similarity between the aftereffects we observed with emotional action adaptation and our understanding of how faces (Haxby et al., 2000; Ellamil et al., 2008; Campbell and Burke, 2009) and voices (Belin et al., 2004) are processed suggest that similar, parallel processing systems might underlie the coding of emotion and identity information from these different social signals. Action representation within the STS can be modulated by emotional body language (Grèzes et al., 2007), and adaptation within this region (cf. Grossman et al., 2010) might underlie the emotional action aftereffect showing identity-independence. The neural substrates underlying our ability to recognize actor identity from body form and motion have not been well delineated, but may involve the Extrastriate Body Area (EBA; Saxe et al., 2006; Urgesi et al., 2007) along with regions in the anterior temporal lobe (although see Downing and Peelen, 2011). Adaptation in more anterior regions of the temporal cortex that takes into account actor identity may underlie the emotional action aftereffect that is influenced by the identity of the individual actors; although this suggestion is more speculative. Anterior cortical regions do not show the same adaptation characteristics as the more posterior cortical regions (cf. Verhoef et al., 2008). The less characteristic longer lasting adaptation effects observed here, and also seen in some face adaptation experiments (e.g., Carbon and Ditye, 2011, 2012), may result from the effects of stimulus repetition on later stages of processing inducing a longer term recalibration of the representation of individuals. Such long lasting aftereffects may shift the observer's reference point according to their recent experience in order to optimize the sensory processing of the external world over a longer period of time.

## Conclusions

This study has demonstrated that prior exposure to emotional whole-body actions influences the perception of the emotion conveyed by subsequent actions via a perceptual adaptation mechanism. Our visual adaptation experiments reveal two separate processing mechanisms for emotional actions with different characteristics: one mechanism that processes actor emotion irrespective of actor identity, and one that processes actor emotion taking into account actor identity. This organization parallels recent data on the processing of face information that suggest that rather than completely separate processing of emotion and identity, representations of emotion, and identity can interact. These mechanisms we study here would not only help us to determine the emotions of individuals around us from their actions and behavior, but also critically ensure the identity of the individual is linked to the specific emotions expressed.

## Author contributions

JW, TJ, and NB designed the research, JW, and JI carried out data collection, JW, JI, TJ and NB analyzed the data, and JW, TJ, and NB wrote the paper.

## Funding

This research was supported by the Economic and Social Research Council (grant number RES-062-23-2797) awarded to NB; JW was supported by an 80th Anniversary Scholarship from the University of Hull. We thank Andy Young and Bruce Keefe for comments on an earlier draft of this manuscript.

## Data access statement

All data supporting this study are provided as supplementary information accompanying this paper.

### Conflict of interest statement

The authors declare that the research was conducted in the absence of any commercial or financial relationships that could be construed as a potential conflict of interest.
