# Supplementary material for: Emotional Actions Are Coded via Two Mechanisms: With and without Identity Representation
Source: Front Psychol. 2016 May 11;7:693. doi: 10.3389/fpsyg.2016.00693 (PMC4862987; doi:10.3389/fpsyg.2016.00693)
Supplement: Supplementary file 1 [file DataSheet1.docx]

Appendix

**Table 1.**

*95% Confidence Interval for 3-way interaction (adaptation emotion x stimulus similarity x response option, ANOVA) reported for the results in Experiment 2 – Control for change in response criterion*

| Identity | Emotion | Response option | 95 % Confidence Interval | |
| --- | --- | --- | --- | --- |
|  |  |  | Lower Bound | Upper Bound |
| Same | Happy | Happy | -26.74 | -7.00 |
|  |  | Sad | 8.74 | 25.43 |
|  |  | Surprised | -2.92 | 5.00 |
|  |  | Disgusted | -4.73 | 3.06 |
|  | Sad | Happy | 3.16 | 23.92 |
|  |  | Sad | -21.92 | -9.75 |
|  |  | Surprised | -3.61 | 5.69 |
|  |  | Disgusted | -4.53 | 7.88 |
| Different | Happy | Happy | -18.55 | 2.29 |
|  |  | Sad | 3.60 | 21.39 |
|  |  | Surprised | -4.55 | 0.81 |
|  |  | Disgusted | -5.78 | 0.78 |
|  | Sad | Happy | -7.77 | 13.19 |
|  |  | Sad | -8.42 | 6.75 |
|  |  | Surprised | -4.16 | 2.91 |
|  |  | Disgusted | -6.28 | 4.62 |

**Table 2.**

*95% Confidence Interval for 2-way interaction (adaptation repeats x ISI duration, ANOVA) reported for the results in Experiment 3a – Dynamics of emotional action aftereffects (Same identity)*

| Adaptation | ISI | 95 % Confidence Interval | |
| --- | --- | --- | --- |
| repeats | Duration (ms) | Lower Bound | Upper Bound |
| 1 | 200 | -0.44 | -0.16 |
|  | 400 | -0.37 | -0.06 |
|  | 800 | -0.40 | -0.11 |
|  | 1600 | -0.38 | -0.14 |
| 2 | 200 | -0.45 | -0.13 |
|  | 400 | -0.44 | -0.16 |
|  | 800 | -0.39 | -0.05 |
|  | 1600 | -0.47 | -0.20 |
| 4 | 200 | -0.43 | -0.11 |
|  | 400 | -0.52 | -0.26 |
|  | 800 | -0.43 | -0.21 |
|  | 1600 | -0.47 | -0.13 |
| 8 | 200 | -0.54 | -0.30 |
|  | 400 | -0.58 | -0.29 |
| 1 | 800 | -0.44 | -0.16 |
|  | 1600 | -0.37 | -0.06 |

**Table 3.**

*95% Confidence Interval for the main effect of adaptation repeats (2x2 ANOVA adaptation repeats x ISI duration) reported for the results in Experiment 3a – Dynamics of emotional action aftereffects (Same identity)*

| Adaptation | 95 % Confidence Interval | |
| --- | --- | --- |
| repeats | Lower Bound | Upper Bound |
| 1 | -0.37 | -0.14 |
| 2 | -0.42 | -0.16 |
| 4 | -0.43 | -0.21 |
| 8 | -0.52 | -0.28 |

**Table 4.**

*95% Confidence Interval for the main effect of ISI duration (2x2 ANOVA adaptation repeats x ISI duration) reported for the results in Experiment 3a – Dynamics of emotional action aftereffects (Same identity)*

| ISI | 95 % Confidence Interval | |
| --- | --- | --- |
| Duration (ms) | Lower Bound | Upper Bound |
| 200 | -0.44 | -0.19 |
| 400 | -0.45 | -0.22 |
| 800 | -0.39 | -0.17 |
| 1600 | -0.45 | -0.21 |

**Table 5.**

*95% Confidence Interval for 2-way interaction (adaptation repeats x ISI duration, ANOVA) reported for the results in Experiment 3b – Dynamics of emotional action aftereffects (Different identity)*

| Adaptation | ISI | 95 % Confidence Interval | |
| --- | --- | --- | --- |
| repeats | Duration (ms) | Lower Bound | Upper Bound |
| 1 | 400 | -0.18 | 0.08 |
|  | 800 | -0.21 | 0.08 |
|  | 1800 | -0.21 | 0.07 |
|  | 3200 | -0.17 | 0.09 |
| 2 | 400 | -0.30 | -0.02 |
|  | 800 | -0.25 | 0.02 |
|  | 1800 | -0.20 | 0.07 |
|  | 3200 | -0.26 | -0.03 |
| 4 | 400 | -0.36 | -0.11 |
|  | 800 | -0.26 | -0.01 |
|  | 1800 | -0.27 | -0.03 |
|  | 3200 | -0.20 | 0.05 |
| 8 | 400 | -0.40 | -0.21 |
|  | 800 | -0.31 | -0.08 |
| 1 | 1800 | -0.37 | -0.09 |
|  | 3200 | -0.30 | 0.00 |

**Table 6.**

*95% Confidence Interval for the main effect of adaptation repeats (2x2 ANOVA adaptation repeats x ISI duration) reported for the results in Experiment 3b – Dynamics of emotional action aftereffects (Different identity)*

| Adaptation | 95 % Confidence Interval | |
| --- | --- | --- |
| repeats | Lower Bound | Upper Bound |
| 1 | -0.16 | 0.05 |
| 2 | -0.21 | -0.03 |
| 4 | -0.24 | -0.05 |
| 8 | -0.32 | -0.12 |

**Table 7.**

*95% Confidence Interval for the main effect of ISI duration (2x2 ANOVA adaptation repeats x ISI duration) reported for the results in Experiment 3b – Dynamics of emotional action aftereffects (Different identity)*

| ISI | 95 % Confidence Interval | |
| --- | --- | --- |
| Duration (ms) | Lower Bound | Upper Bound |
| 400 | -0.28 | -0.09 |
| 800 | -0.23 | -0.02 |
| 1600 | -0.23 | -0.03 |
| 3200 | -0.20 | 0.01 |
